# Hepatitis A Vaccination Coverage Among People With Chronic Liver Disease in England (HEALD): Protocol for a Retrospective Cohort Study

**DOI:** 10.2196/51861

**Published:** 2023-10-24

**Authors:** Bernardo Meza-Torres, Anna Forbes, William Elson, Debasish Kar, Gavin Jamie, William Hinton, Xuejuan Fan, Rachel Byford, Michael Feher, Martin Whyte, Mark Joy, Simon de Lusignan

**Affiliations:** 1 Clinical Informatics and Health Outcomes Research Group Nuffield Department of Primary Care Health Sciences University of Oxford Oxford United Kingdom; 2 School of Biosciences and Medicine University of Surrey Guildford United Kingdom; 3 Royal College of General Practitioners Research and Surveillance Centre London United Kingdom

**Keywords:** chronic liver disease, computerized, data accuracy, data extract, ethnicity, fatty liver disease, general practitioner, hepatitis A vaccination, hepatitis, liver disease, medical record systems, primary care, routine data sets, Systematized Nomenclature of Medicine, vaccination monitoring and surveillance, vaccination

## Abstract

**Background:**

Hepatitis A outbreaks in the United Kingdom are uncommon. Most people develop mild to moderate symptoms that resolve, without sequelae, within months. However, in high-risk groups, including those with underlying chronic liver disease (CLD), hepatitis A infection can be severe, with a higher risk of mortality and morbidity. The Health Security Agency and the National Institute of Health and Care Excellence recommend preexposure hepatitis A vaccination given in 2 doses to people with CLD, regardless of its cause. There are currently no published reports of vaccination coverage for people with CLD in England or internationally.

**Objective:**

This study aims to describe hepatitis A vaccination coverage in adults with CLD in a UK primary care setting and compare liver disease etiology, sociodemographic characteristics, and comorbidities in people who are and are not exposed to the hepatitis A vaccine.

**Methods:**

We will conduct a retrospective cohort study with data from the Primary Care Sentinel Cohort of the Oxford-Royal College of General Practitioners Clinical Informatics Digital Hub database, which is nationally representative of the English population. We will include people aged 18 years and older who have been registered in general practices in the Research and Surveillance Centre network and have a record of CLD between January 1, 2012, and December 31, 2022, including those with alcohol-related liver disease, chronic hepatitis B, chronic hepatitis C, nonalcohol fatty liver disease, Wilson disease, hemochromatosis, and autoimmune hepatitis. We will carefully curate variables using the Systematized Nomenclature of Medicine Clinical Terms. We will report the sociodemographic characteristics of those who are vaccinated. These include age, gender, ethnicity, population density, region, socioeconomic status (measured using the index of multiple deprivation), obesity, alcohol consumption, and smoking. Hepatitis A vaccination coverage for 1 and 2 doses will be calculated using an estimate of the CLD population as the denominator. We will analyze the baseline characteristics using descriptive statistics, including measures of dispersion. Pairwise comparisons of case-mix characteristics, comorbidities, and complications will be reported according to vaccination status. A multistate survival model will be fitted to estimate the transition probabilities among four states: (1) diagnosed with CLD, (2) first dose of hepatitis A vaccination, (3) second dose of hepatitis A vaccination, and (4) death. This will identify any potential disparities in how people with CLD get vaccinated.

**Results:**

The Research and Surveillance Centre population comprises over 8 million people. The reported incidence of CLD is 20.7 cases per 100,000. International estimates of hepatitis A vaccine coverage vary between 10% and 50% in this group.

**Conclusions:**

This study will describe the uptake of the hepatitis A vaccine in people with CLD and report any disparities or differences in the characteristics of the vaccinated population.

**International Registered Report Identifier (IRRID):**

PRR1-10.2196/51861

## Introduction

### Overview

Hepatitis A (Hep A) is a viral infection of the liver caused by the hepatitis A virus (HAV). HAV is transmitted through the fecal-oral route, mainly by exposure to contaminated water sources. Hep A outbreaks are uncommon in the United Kingdom, and most people manifest mild to moderate symptoms that tend to resolve, without sequelae, within several months. In high-risk groups, however, such as people with an underlying chronic liver disease (CLD), a superimposed acute infection with HAV can be severe, potentially requiring hospitalization and carrying a higher risk of death. According to the World Health Organization, approximately 100 million Hep A infections occur globally per year, resulting in 15,000 to 30,000 deaths per year [1]. Of these, the number of people with a preexisting CLD diagnosis remains unclear.

CLD largely results from the progressive destruction and regeneration of hepatocytes, leading to fibrosis and cirrhosis, and has a range of etiologies. The most common causes of CLD include alcohol-related liver disease (ALD), chronic hepatitis B (CH-B) infection, chronic hepatitis C (CH-C) infection, and nonalcoholic fatty liver disease (NAFLD), the latter being associated with metabolic syndrome and obesity [2,3]. Deaths in the United Kingdom from CLD have increased annually for the past decade, peaking at 20.6 per 100,000 population in 2020 [4,5], and are expected to surpass the numbers of premature deaths from coronary heart disease [3], CLD-related hospitalizations in the United Kingdom have increased by half, contributing to the £2.1 (US $2.62) billion per year currently spent on treating liver disease [4,6].

The UK’s public health body, the Health Security Agency, and the national guideline body, the National Institute for Health and Care Excellence, recommend preexposure Hep A vaccination to all people with CLD, regardless of the cause [7,8]. The Hep A vaccine series consists of 2 doses, with the second dose at least 6 months after the first.

The data from the United States have shown that less than one-third of at-risk adults receive any vaccination for Hep A, and only one-fifth receive both doses [2,9-12]. Adherence to the vaccination schedule was worse at the extremes of adult age, in ethnic minorities, and in those of low socioeconomic status [10]. The same is true in the United Kingdom; under 35% of eligible people receive the complete Hep A vaccination course, though these data are presented for all eligible patients, regardless of CLD status [13].

Hence, there are no published reports of Hep A vaccination coverage in people with CLD. The purpose of this study is to report Hep A vaccine coverage in people with CLD in UK primary care, with a focus on whether there are disparities as to which people with CLD receive Hep A vaccination.

### Aims

The aim of this study is to report Hep A vaccination coverage in people with CLD in English primary care and the predictors of receiving a single dose or full vaccination course.

### Objectives

The objectives of this study are as follows:

1. To report yearly Hep A vaccination coverage in people with CLD by:
  sociodemographic characteristics (eg, age, sex, ethnicity, deprivation, BMI category, smoking status, and alcohol consumption)
  etiology of CLD (eg, ALD, CH-B, CH-C, and NAFLD).
  CLD complications (eg, renal disease, ascites, liver failure and transplant, and other cardiometabolic diseases, including diabetes and heart disease)
  comorbidities and exposures (eg, Cambridge Multimorbidity Score [CMMS], bile duct and colon cancers, at least three doses of COVID-19 vaccine, and flu vaccination).
2. To report the predictors of 1 and 2 doses of Hep A vaccination in people with CLD.

## Methods

### Study Design

This is a retrospective cohort study using data from the nationally representative Primary Care Sentinel Cohort (PCSC) of the Oxford-Royal College of General Practitioners (RCGP) Research and Surveillance Centre (RSC) [14]. The RSC is one of Europe’s oldest sentinel systems, and hepatitis is one of its 32 monitored conditions [15]. The database has previously been used for liver disease and hepatitis research [3,16].

We will use electronic health record data for the period between January 1, 2012, and December 31, 2022. Data will be extracted retrospectively at the time of the beginning of the study.

### Setting

#### Data Source

Data will be extracted from the PCSC of the RSC. The PCSC consists of a nationally representative sample of 7.4 million patients registered in primary care practices in England [17,18]. Pseudonymized data will be extracted from the secure environment of the Oxford-RCGP Clinical Informatics Digital Hub (ORCHID) [19].

#### CLD Ontology and Curated Variables

Key population characteristics, index conditions, and outcomes will be identified using the Systematized Nomenclature of Medicine Clinical Terms (SNOMED CT), in accordance with NHS Digital [20]. To identify these, we will curate data to ensure its use in future studies, supporting the findable, accessible, interoperable, and reusable open science principles [21].

The preliminary SNOMED CT codes to be used to identify CLD as an index condition are summarized in Multimedia Appendix 1. A conceptual model and suitable ontology are developed before carrying out the data extraction [22,23]. The identification of CLD-related concepts will be based on the clinical diagnoses as recorded in primary care; the researchers will not develop ad hoc diagnostic criteria. Clinical codes for CLD will be grouped by their main etiology; a hierarchical ontological approach using the conceptual model will be followed if overlapping concepts are encountered.

#### Data Quality

The RSC has over 55 years of experience in infectious disease surveillance, including vaccination coverage and effectiveness studies [18,24,25]. Viral hepatitis is one of the RSC’s monitored conditions.

### Study Population

We will include adults registered in general practices within the RSC network with a diagnosis of CLD, as defined by our ontology, within the period between January 1, 2012, and December 31, 2022. The following inclusion criteria will be applied: (1) people registered in the RSC general practices, aged 18 years and older, with a diagnosis of CLD; and (2) the ontology for CLD includes ALD, CH-B, CH-C, NAFLD, Wilson Disease, hemochromatosis, autoimmune hepatitis, primary sclerosing cholangitis, and primary biliary cholangitis [2-4].

The following exclusion criteria are applied: (1) patients with recorded contraindications to the Hep A vaccine (confirmed anaphylactic reaction to a previous dose of Hep A containing vaccine or to any of its components); and (2) records of administration of postinfection or risk-of-infection immunoglobulins.

### Variables

#### Overview

The study population is those with CLD as an index condition, as defined in the inclusion criteria. The variables will be curated for the identification of CLD-related codes in the computerized medical records of adults registered in the RSC network (Multimedia Appendix 1).

Comparisons will be conducted between population subgroups with different CLD etiologies, sociodemographic characteristics, complications, and comorbidity profiles.

The outcome of the first objective will be the coverage rates of Hep A vaccination among those with CLD.

For the second objective, we will explore significant independent predictors of the following outcomes among those with CLD: (1) one dose of Hep A vaccination and (2) two doses of Hep A vaccination.

Hep A vaccination, as defined by the UK vaccination scheme, includes an initial 0.5 mL for 1 dose, followed by a booster dose of 0.5 mL after 6-12 months. The booster dose may be delayed by up to 3 years if not given after the recommended interval following the primary dose [8]. For this study, we will report boosters administered within 12 months of the first dose for the base case analysis and 3 years from the first dose for a sensitivity analysis. Hep A vaccination in the United Kingdom is available as a monovalent vaccine, as a combined Hep A and hepatitis B vaccine, and as a Hep A and typhoid vaccine [7]. We will consider any Hep A vaccination type as an outcome.

#### Covariates

Sociodemographic characteristics will be reported closest to the earliest date of CLD diagnosis as baseline, within a 10-year range for time-sensitive variables. These include age, gender, ethnicity (categorized into White, Black, Asian, multiracial, and other), rurality (measure of population density), region, socioeconomic status (measured using the index of multiple deprivation), obesity, alcohol consumption, and smoking. A history of comorbid conditions and complications will be extracted from 10 years of computerized medical records, including osteoporosis, bile duct and colon cancers, renal disease (including calculi), ascites, liver failure and transplant, and cardiometabolic disease. The CMMS will be used to report on comorbidity profiles [26,27].

### Study Size

Age-standardized incidence rates for CLD worldwide are reported as 20.7/100,000 [28]. The RSC network comprises a nationally representative sample of 7.4 million registered patients. This brings an estimate of up to 1656 potential patients with CLD per year of study to be included in our final study population. This is consistent with previous reports on the CLD population in the RSC network [29].

In the United States, Hep A vaccination coverage is reported to range between 10% and 50% [2,9]. Therefore, we expect between 165 and 828 individuals to be found to have both the index condition and outcome of interest per year for a 10-year study period.

**Figure 1:**
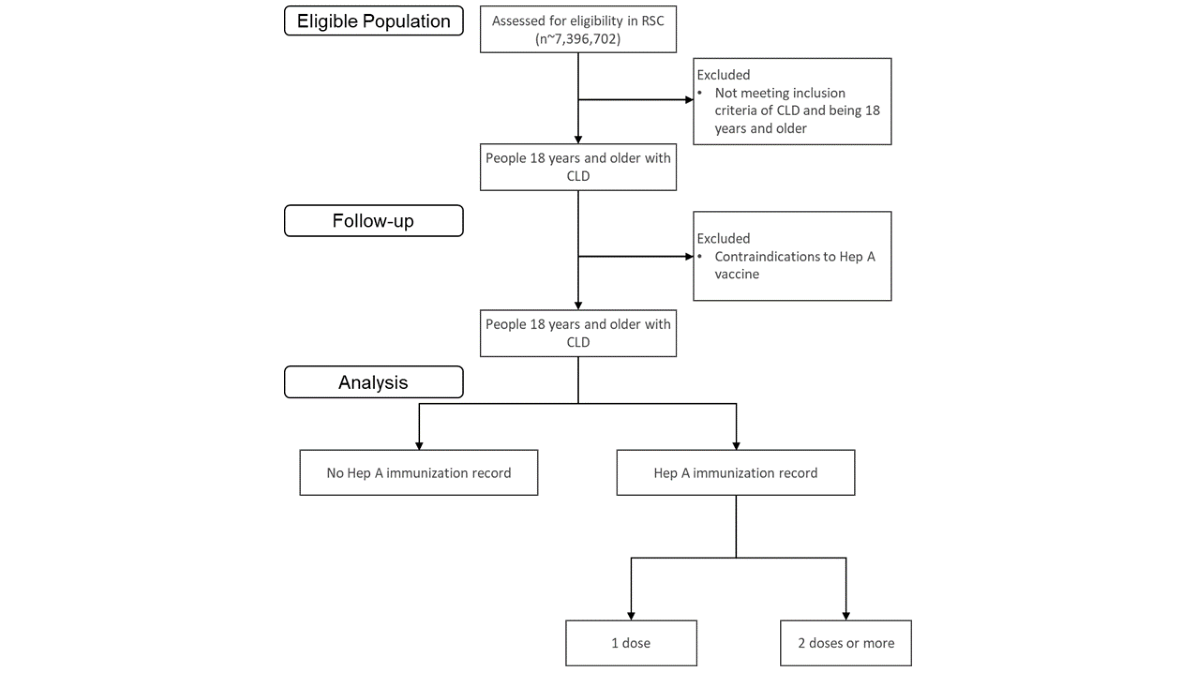
Consolidated Standards of Reporting Trials diagram for eligible individuals in the Research and Surveillance Centre (RSC) network for a retrospective cohort analysis of adults with chronic liver disease (CLD) in England between January 1, 2012, and December 31, 2022, evaluating hepatitis A vaccination with 1 and 2 doses as outcomes.

The study population will be categorized and stratified for analysis following the CONSORT (Consolidated Standards of Reporting Trials) diagram (Figure 1).

### Statistical Analysis

#### Reporting Hep A Vaccination Coverage in People With CLD

Hep A vaccination rates will be calculated using the number of vaccinated people with CLD as the numerator and estimates of the CLD population in the ORCHID data set as the denominator. Baseline characteristics of each study group (eg, age and gender) will be summarized using descriptive statistics, including measures of dispersion (eg, SDs and IQRs).

To compare individual characteristics by vaccination status (eg, CLD disease type, sociodemographic characteristics, complications, and comorbidities), we will use descriptive statistics, with pairwise comparisons using the chi-square or Fisher exact tests for categorical variables and the *t* test or Wilcoxon test for continuous variables. If stratification involves more than 2 groups, differences across groups will be tested using the Kruskal-Wallis test for continuous variables and the Pearson chi-square test for categorical variables, followed by pairwise comparisons as above described. All statistical tests will be 2-sided with *P*<.05 considered statistically significant.

#### Reporting Predictors of 1 and 2 Doses of Hep A Vaccination in People With CLD

##### Overview

**Figure 2:**
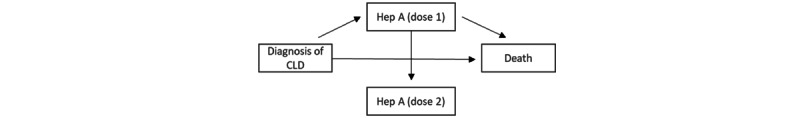
State transition diagram for competing risks multistate model for a retrospective cohort of adults with chronic liver disease (CLD) in England between January 1, 2012, and December 31, 2022, evaluating hepatitis A (Hep A) vaccination with 1 and 2 doses as outcomes and death as a competing risk represented as an absorbing state.

A multistate model will be used on a retrospective cohort of individuals identified in the ORCHID diagnosed with CLD. People enter the study on the first diagnosis of CLD, and we will model the transition probabilities between the identified states: diagnosed with CLD, first dose of Hep A vaccination, second dose of Hep A vaccination, and death (Figure 2).

A multistate survival model will be fitted to estimate the transition probabilities between the 4 identified study states (Figure 2). The states are (1) diagnosis of CLD, (2) first dose of Hep A vaccination, (3) death, and (4) second dose of Hep A vaccination. The outcome of interest is the Hep A vaccination. Therefore, the state of a second dose of Hep A vaccination is a censored state, with no further transitions to death; the moment a patient receives 2 doses of Hep A vaccination, the follow-up ends. Death is an absorbing state, with no further transitions allowed. Death is included as a competing risk for vaccination, not as an outcome. This means that when calculating the transition probabilities to vaccination, we adjust for when a person is not vaccinated because of death before vaccination rather than for any other predictors of nonvaccination.

Patients lost to follow-up will be right censored and evaluated accordingly as per the likelihood function of the “msm” R package [30].

Multistate probabilities are based on the same assumptions as a Markov process:

1. The health states considered in the model are thorough and mutually exclusive.
2. The probability to move from one health state to following states only depends on (is conditioned on) the health state of the individual at transition time (memoryless process).
3. Transition probabilities are steady over time.

The state transition probabilities will be estimated conditional on the study risk factors. Study risk factors include (1) etiology of CLD (eg, ALD, CH-B, CH-C, and NAFLD); (2) sociodemographic characteristics (eg, age, sex, ethnicity, deprivation, obesity, smoking, and alcohol consumption); (3) CLD complications (eg, bile duct and colon cancers, renal disease, ascites, liver failure and transplant, and cardiometabolic disease); and (4) comorbidities (eg, CMMS, bile duct and colon cancers, at least 3 doses of COVID-19 vaccine, and flu vaccination).

The multistate model will be estimated using the “msm” (version 1.6.9) package in R [31].

##### Missing Data

Descriptive analyses will be conducted on the overall study population and applicable subgroups. Data that are not documented in our database will be reported as missing.

Missing data will be presented as a separate category in univariate statistics and compared to the reference category through univariate odds ratios. Missing data categories will be imputed to the reference category if no significant differences are found in the reference category. Missing data categories will be otherwise included in the analysis as an independent category if evidence suggests they are not missing at random; for example, significant differences to the reference category exist.

##### Sensitivity Analysis

We will estimate 2 multistate models by varying the definition of Hep A dose 2. In the first model, the second dose must be administered within 1 year of the first dose; in the second model, dose 2 may occur within 3 years of the first administered dose. A 3-year time window between vaccinations is likely to exclude those who have been recently vaccinated. Hence the need for such a sensitivity analysis. A complete-case analysis to assess any differences due to missing data can be conducted after assessing the missingness of the descriptive data.

##### Spurious Data

Data quality (eg, consistency and accuracy) will be assessed at the point of data extraction through an outlier analysis comparing the covariate’s values to plausible biological measures. Spurious values will be categorized as missing values and handled according to the methodology layout.

### Ethical Considerations

Anonymized electronic health record data were accessible for research purposes following ethical approval from the Central University Research Ethics Committee at the University of Oxford (under reference R80951/RE001) for human participant research. Anonymized patient data were retrieved from electronic health record providers from those users who had consented for their data to be used for secondary research purposes. No form of compensation was provided to participants. Generative artificial intelligence was not used in any portion of the manuscript.

## Results

The primary care cohort comprised 1704 practices and 7,396,702 registered patients between the January 1, 2012, and December 31, 2022 (Figure 1). We have created an initial clinical code ontology using existing SNOMED CT–curated variables for our sociodemographic variables, hepatitis, and CLD by etiology (Multimedia Appendix 1). The ontology will be expanded to include paraclinical indicators of CLD to identify cases where an explicit diagnosis or specific etiology has not been recorded. Other relevant covariates will be created as required for the study.

The phases of this study will include curating the required variables, conducting our data extraction, statistical analysis, and reporting of the findings through scientific publications.

## Discussion

### Overview

This study will inform on the state of preventive Hep A vaccination for people with CLD in a representative primary care sentinel network in England. From a total of 7.4 million patients in the RSC network, we will report on the sociodemographic and clinical characteristics of people with CLD with and without Hep A vaccination, for 1 and 2 doses.

The multistate model, adjusted for study risk factors, will identify those associated with having the vaccination. This will inform on the individual characteristics that are associated with an immunization, informing on the subgroups that could benefit from higher engagement with preventive primary care. Any disparities identified could be used to target clinical and public health interventions to improve vaccination coverage in this at-risk group.

### Comparisons With Previous Work

The results will be compared to previous reports on preexposure Hep A vaccination coverage rates of 10%-50% in the United States [2,9,10,12]. For the United Kingdom, there are no published reports on vaccination coverage in this subpopulation, to the best of our knowledge.

### Limitations

Selection and misclassification biases can be associated with the retrospective study design. However, this can be mitigated by the availability of reliable records in the database. Similarly, the retrospective study design has a limitation to establishing causal relationships due to the temporality and lack of randomization. However, retrospective associations can be a timely source of epidemiological data to guide further research.

There exists potential confounding due to case-mix characteristics, which will be mitigated through multivariate time-to-event modeling in the multistate model.

Large general practice databases are susceptible to coding errors or inaccuracies that may be associated with ascertainment and misclassification biases. This is mitigated by the large representative sample of UK practices within the RSC network, which controls for random error and variation on these parameters [18].

### Conclusions

This descriptive epidemiological study will fill an important gap in our knowledge about Hep A vaccination in people with CLD in the United Kingdom by describing the uptake of Hep A vaccine in people with CLD and reporting any disparities or differences in characteristics of the vaccinated population.

### Data Handling and Record Keeping

Data are held on dedicated, secure servers at the RCGP data and analytics hub in the Clinical Informatics and Health Outcomes Research Group, based at the Nuffield Department of Primary Care Health Sciences, University of Oxford. The research group’s secure network is sited behind a firewall within the university’s network; all inbound connections are blocked, but outbound connections are allowed. Only staff members or associated members of the research group approved by the head of department can access the data from a virtual desktop (on the ORCHID secure server) using a University of Oxford computer and their unique access credentials. The use of personal equipment is not permitted and cannot be connected to a secure network. Pseudonymized study data will be archived for at least 5 years.

## Appendix

Multimedia Appendix 1 Preliminary list of SNOMED CT codes used to identify individuals with chronic liver disease registered in the RSC network in England between the 1st January 2012 and the 31st December 2022.

## Abbreviations

ALD: alcohol-related liver disease
CH-B: Chronic hepatitis B
CH-C: Chronic hepatitis C
CLD: chronic liver disease
CMMS: Cambridge Multimorbidity Score
CONSORT: Consolidated Standards of Reporting Trials
HAV: hepatitis A virus
Hep A: hepatitis A
NAFLD: nonalcoholic fatty liver disease
ORCHID: Oxford-RCGP Clinical Informatics Digital Hub
PCSC: Primary Care Sentinel Cohort
RCGP: Royal College of General Practitioners
RSC: Research and Surveillance Centre
SNOMED CT: Systematized Nomenclature of Medicine Clinical Terms

## Data Availability

Apart from necessary access by the research team, direct access may be granted to authorized representatives from the sponsor or host institutions for monitoring or audit of the study to ensure compliance with regulations.

Researchers will maintain a log of data gathering using study IDs rather than personal identifying information.
