# Supplementary material for: Hepatitis A Vaccination Coverage Among People With Chronic Liver Disease in England (HEALD): Protocol for a Retrospective Cohort Study
Source: JMIR Res Protoc. 2023 Oct 24;12:e51861. doi: 10.2196/51861 (PMC10630863; doi:10.2196/51861)
Supplement: Multimedia Appendix 1 [file resprot_v12i1e51861_app1.pdf]

Concept description	SNOMED CT	Aetiology description
Acute hepatitis B with delta-agent (coinfection) without hepatic coma	186626002	Infective
Acute hepatitis B with hepatic coma	424099008	Infective
Chronic hepatitis B co-occurrent with hepatitis C and hepatitis D	838380002	Infective
Acute hepatitis B with hepatic coma	424099008	Infective
Chronic hepatitis C	128302006	Infective
Chronic hepatitis C caused by Hepatitis C virus genotype 1	768127002	Infective
Chronic hepatitis C caused by Hepatitis C virus genotype 1a	768289009	Infective
Chronic hepatitis C caused by Hepatitis C virus genotype 1b	768288001	Infective
Chronic hepatitis C caused by Hepatitis C virus genotype 2	768125005	Infective
Chronic hepatitis C caused by Hepatitis C virus genotype 3	768006009	Infective
Chronic hepatitis C caused by Hepatitis C virus genotype 4	768126006	Infective
Chronic hepatitis C caused by hepatitis C virus genotype 5	767810006	Infective
Chronic hepatitis C caused by hepatitis C virus genotype 6	767809001	Infective
Chronic hepatitis C co-occurrent with human immunodeficiency virus infection	838377003	Infective
Chronic hepatitis C with stage 2 fibrosis	703866000	Infective
Chronic hepatitis C with stage 3 fibrosis	347891000119103	Infective
Chronic hepatitis E	450880008	Infective
Chronic infectious pancreatitis	838375006	Infective
Chronic active hepatitis C	708198006	Infective
Chronic active type B viral hepatitis	50167007	Infective
Chronic active viral hepatitis	66870002	Infective
Chronic aggressive type B viral hepatitis	1116000	Infective
Chronic aggressive viral hepatitis	89789003	Infective
Bacterial portal cirrhosis	197303009	Infective
Anicteric type B viral hepatitis	53425008	Infective
Syphilis of liver	86028001	Infective
Syphilitic cirrhosis	16070004	Infective
Syphilitic portal cirrhosis	197305002	Infective
Tropical calcific chronic pancreatitis	724540009	Infective
Tuberculosis of liver	186273003	Infective
Type B viral hepatitis	66071002	Infective
Unilobular portal cirrhosis	197291001	Infective
Chronic hepatitis B with hepatic coma	424340000	Infective
Chronic hepatitis B with hepatic coma	424340000	Infective
Viral hepatitis B without hepatic coma	111891008	Infective
Viral hepatitis C	50711007	Infective
Viral hepatitis D	707341005	Infective
Viral hepatitis E	7111000119109	Infective
Chronic hepatitis B with hepatic coma	424340000	Infective
Viral hepatitis, type G	397575003	Infective
Dilated cardiomyopathy secondary to haemochromatosis	8209004	Metabolic
Dilated cardiomyopathy secondary to haemochromatosis	8209004	Metabolic
Hepatic coma due to acute hepatic failure	1082511000119100	Metabolic
Hepatic coma due to acute hepatic failure	1082511000119100	Metabolic
Hepatic coma due to acute hepatitis B with delta agent	186624004	Infective
Reactivation of hepatitis B viral hepatitis	446698005	Infective
Reactivation of hepatitis C viral hepatitis	702969000	Infective
Relapsing viral hepatitis	58282009	Infective
Congenital viral hepatitis B infection	60498001	Infective
HIV-related sclerosing cholangitis	281388009	Infective
Non-A, non-B, non-C hepatitis	406584008	Infective
Occult chronic type B viral hepatitis	713966008	Infective
Secondary syphilis of liver	80770009	Infective
Hepatic coma due to acute hepatitis B with delta agent	186624004	Infective
Hepatic coma due to acute hepatitis B with delta agent	186624004	Infective
Hepatic coma due to acute hepatitis C	125921000119106	Infective
Hepatic coma due to acute hepatitis C	125921000119106	Infective
Hepatic coma due to acute hepatitis C	125921000119106	Infective
Hepatic coma due to alcoholic liver failure	1082621000119100	Alcoholic
Hepatic coma due to alcoholic liver failure	1082621000119100	Alcoholic
Hepatic coma due to chronic hepatic failure	1085091000119100	Metabolic
Hepatic coma due to chronic hepatic failure	1085091000119100	Metabolic
Hepatic coma due to chronic hepatitis B with delta agent	153091000119109	Infective
Hepatic coma due to chronic hepatitis B with delta agent	153091000119109	Infective
Hepatic coma due to chronic hepatitis B with delta agent	153091000119109	Alcoholic
Hepatic coma due to chronic hepatitis C	146371000119104	Infective
Hepatic coma due to chronic hepatitis C	146371000119104	Infective
Hepatic coma due to chronic hepatitis C	146371000119104	Alcoholic
Hepatic coma due to hepatitis	103931000119102	Infective
Hepatic coma due to hepatitis	103931000119102	Alcoholic
Hepatic coma due to subacute liver failure	1092691000119100	Metabolic
Chronic pancreatitis due to acute alcohol intoxication	767291004	Alcoholic
Chronic pancreatitis due to chronic alcoholism	154211000119108	Alcoholic
Fibrosis of liver caused by alcohol	721710005	Alcoholic
Oesophageal varices in alcoholic cirrhosis of the liver	309783001	Alcoholic
Hepatic coma due to subacute liver failure	1092691000119100	Metabolic
Hepatic coma due to viral hepatitis C	186628001	Infective

Hepatic coma due to viral hepatitis C	186628001	Infective
Hepatic coma due to viral hepatitis C	186628001	Infective
Hepatic encephalopathy	13920009	Metabolic
Toxic liver disease with chronic active hepatitis	197361008	Autoimmune
Toxic liver disease with chronic lobular hepatitis	197360009	Autoimmune
Toxic liver disease with chronic persistent hepatitis	197359004	Autoimmune
Toxic liver disease with fibrosis and cirrhosis of liver	197362001	Autoimmune
Toxic liver disease with hepatic necrosis	197356006	Autoimmune
Toxic portal cirrhosis	197301006	Autoimmune
Chronic liver disease	328383001	Autoimmune
Chronic lobular hepatitis	57339008	Autoimmune
Chronic lymphocytic cholangitis-cholangiohepatitis	9843006	Autoimmune
Chronic necrosis of liver	863957008	Autoimmune
Chronic passive congestion of liver	34736002	Autoimmune
Chronic persistent hepatitis	41889008	Autoimmune
Chronic nonalcoholic liver disease	79720007	Autoimmune
Chronic pancreatitis	235494005	Autoimmune
Fatty portal cirrhosis	197294009	Autoimmune
Ferro-cerebro-cutaneous syndrome	774151000	Autoimmune
Fibropolycystic disease of liver	737202006	Autoimmune
Inflammatory disease of liver	128241005	Autoimmune
Lupus hepatitis	19682006	Autoimmune
Hepatic ascites co-occurrent with chronic active hepatitis due to toxic liver disease	1092801000119100	Autoimmune
Liver abscess and sequelae of chronic liver disease	197324004	Autoimmune
Recurrent hepatitis	197286002	Autoimmune
Relapsing pancreatitis	234689009	Autoimmune
Cirrhosis - non-alcoholic	266468003	Cirrhosis
Cirrhosis and chronic liver disease	197279005	Cirrhosis
Cirrhosis associated with cystic fibrosis	776981000000103	Cirrhosis
Cirrhosis of liver	19943007	Cirrhosis
Cirrhosis of liver caused by amiodarone	725939009	Cirrhosis
Cirrhosis of liver caused by methotrexate	725938001	Cirrhosis
Cirrhosis of liver caused by methyldopa	725940006	Cirrhosis
Cirrhosis of liver due to and following cardiac procedure	871619002	Cirrhosis
Cirrhosis of liver due to chronic hepatitis C	831000119103	Cirrhosis
Cirrhosis of liver due to hepatitis B	103611000119102	Cirrhosis
Cirrhosis of liver with primary sclerosing cholangitis	735733008	Cirrhosis
Cirrhosis secondary to cholestasis	271440004	Cirrhosis
Cirrhotic cardiomyopathy	725416005	Cirrhosis
Clonorchiasis with biliary cirrhosis	37688005	Cirrhosis
Decompensated cirrhosis of liver	716203000	Cirrhosis
Diffuse nodular cirrhosis	197293003	Cirrhosis
Early cirrhosis	371139006	Cirrhosis
Florid cirrhosis	76301009	Cirrhosis
Indian childhood cirrhosis	6183001	Cirrhosis
Infectious cirrhosis	235896001	Cirrhosis
Juvenile portal cirrhosis	266471006	Cirrhosis
Laennec's cirrhosis, non-alcoholic	235895002	Cirrhosis
Latent cirrhosis	123716002	Cirrhosis
Macronodular cirrhosis	43904005	Cirrhosis
Micronodular cirrhosis	21861000	Cirrhosis
Hypoxia-associated cirrhosis	235897005	Cirrhosis
Idiopathic copper associated cirrhosis of liver	715864007	Cirrhosis
Multilobular portal cirrhosis	266469006	Cirrhosis
Oesophageal varices in cirrhosis of the liver	308129003	Cirrhosis
Nutritional cirrhosis	123605001	Cirrhosis
Obstructive biliary cirrhosis	109819003	Cirrhosis
Parasitic cirrhosis	33144001	Cirrhosis
North American Indian childhood cirrhosis	699189004	Cirrhosis
NPHP3-related Meckel-like syndrome	773737004	Cirrhosis
Pigment cirrhosis	78208005	Cirrhosis
Pigmentary portal cirrhosis	197299004	Cirrhosis
Pipestem portal cirrhosis	197300007	Cirrhosis
Portal cirrhosis	419728003	Cirrhosis
Secondary biliary cirrhosis	12368000	Cirrhosis
Toxic cirrhosis	123604002	Cirrhosis
Subfulminant hepatic failure	235885009	Cirrhosis
Symmer's pipe-stem fibrosis	240792005	Cirrhosis
Hepatic fibrosis	62484002	Fibrosis
Hepatic fibrosis with hepatic sclerosis	235901004	Fibrosis
Hepatic fibrosis, renal cyst, intellectual disability syndrome	771149000	Fibrosis
Liver disease due to cystic fibrosis	427022004	Fibrosis
Hepatic encephalopathy	13920009	Metabolic
Hepatic glycogen phosphorylase kinase deficiency	297255007	Fibrosis
Hepatic glycogen synthase deficiency	725026008	Fibrosis
Hepatic granulomas in berylliosis	197367007	Fibrosis
Hepatic granulomas in sarcoidosis	197368002	Fibrosis
Hepatic sclerosis	235899008	Fibrosis
Hunter's syndrome, mild form	5667009	Fibrosis
Hunter's syndrome, severe form	73146005	Fibrosis
Idiopathic portal hypertension	722867009	Fibrosis
IgG4-related sclerosing cholangitis	722870008	Fibrosis

Intrahepatic phlebosclerosis and fibrosis	235902006	Fibrosis
Ischaemic hepatitis	235877000	Fibrosis
Nonalcoholic steatohepatitis	442685003	Fibrosis
Pericellular fibrosis of congenital syphilis	235898000	Fibrosis
Periportal fibrosis	870517000	Fibrosis
Local recurrence of malignant tumour of liver	314963000	Fibrosis
Portal fibrosis without cirrhosis	197316009	Fibrosis
Polyglucosan body myopathy type 1	774148007	Fibrosis
Portal and splenic vein sclerosis	235900003	Fibrosis
Reynolds syndrome	715401008	Fibrosis
Sclerosing cholangitis	235917005	Fibrosis
Sclerosis of portal vein and splenic vein caused by antiretroviral drug	713965007	Fibrosis
Secondary sclerosing cholangitis	197442005	Fibrosis
Stage 3 hepatic fibrosis	10690671000119100	Fibrosis
Steatohepatitis	442191002	Fibrosis
Steatosis of liver	197321007	Fibrosis
Septal fibrosis of liver	123607009	Fibrosis
Sequela of chronic liver disease	444918006	Fibrosis
Hepatic veno-occlusive disease with immunodeficiency syndrome	724361001	Fibrosis
Hepatitis B associated with Human immunodeficiency virus infection	442134007	Fibrosis
Hepatomegaly associated with AIDS	421230000	Fibrosis
Hepatosplenic gamma-delta cell lymphoma	699657009	Fibrosis
Hepatosplenic gamma-delta cell lymphoma	103685007	Fibrosis
Hepatosplenic T-cell lymphoma	445406001	Fibrosis
Pulmonary fibrosis, hepatic hyperplasia, bone marrow hypoplasia syndrome	723829000	Fibrosis
Hepatic encephalopathy in fulminant hepatic failure	449901005	Metabolic
Complication of transplanted liver	33167004	Transplant
Exploration of liver transplant	235458006	Transplant
Hyperacute rejection of liver transplant	432772009	Transplant
Heterotopic liver transplant	174426002	Transplant
Liver transplant disorder	235910007	Transplant
Liver transplant failure	235912004	Transplant
Liver transplant failure and rejection	213153001	Transplant
Liver transplant planned	704201006	Transplant
Liver transplant recipient	702777009	Transplant
Liver transplant rejection	235911006	Transplant
Liver transplant with recipient hepatectomy	27280000	Transplant
Liver transplant without recipient hepatectomy	28009009	Transplant
Living donor liver transplantation	853761000000103	Transplant
Orthotopic liver transplant	174425003	Transplant
Orthotopic transplantation of whole liver	426356008	Transplant
Replacement of previous liver transplant	174427006	Transplant
Transplantation of hepatocytes	428198008	Transplant
Transplantation of liver	18027006	Transplant
Transplanted liver present	737297006	Transplant
Congenital absence of bile duct	1287007	Congenital
Congenital absence of hepatic ducts	204781002	Congenital
Congenital absence of liver	3650004	Congenital
Congenital absence of liver and/or gallbladder	204787003	Congenital
Congenital atresia of extrahepatic bile duct	82821008	Congenital
Congenital biliary atresia	77480004	Congenital
Congenital bronchobiliary fistula	719452004	Congenital
Congenital cystic disease of liver	72925005	Congenital
Congenital hepatic fibrosis	79607001	Congenital
Congenital hepatitis C infection	278929008	Congenital
Congenital hepatomegaly	407000	Congenital
Congenital hypoplasia of bile duct	68094008	Congenital
Congenital kink of cystic duct	253808004	Congenital
Congenital non-A non-B hepatitis infection	276668008	Congenital
Congenital obstruction of bile duct	51038004	Congenital
Congenital stricture of bile duct	1512006	Congenital
Congenital stricture of common bile duct	281095009	Congenital
Congenital syphilitic hepatomegaly	192008	Congenital
Congenital tracheobiliary fistula	720394008	Congenital
Cruveilhier-Baumgarten syndrome	45256007	Congenital
Cryptogenic cirrhosis	89580002	Congenital
Danon disease	419097006	Congenital
Contiguous ABCD1 DXS1357E deletion syndrome	773415005	Congenital
Familial arthrogryposis-cholestatic hepatorenal syndrome	62216007	Congenital
Familial chronic pancreatitis	235956004	Congenital
Familial hypercholanemia	723360007	Congenital
Fatal congenital nonlysosomal heart glycogenosis	459062008	Congenital
Intrahepatic biliary atresia	253807009	Congenital
Intrahepatic biliary hypoplasia	276723008	Congenital
Mitochondrial DNA depletion syndrome hepatocerebrorenal form	782771007	Congenital
Mitochondrial DNA depletion syndrome, hepatocerebral form due to DGUOK deficiency	783734000	Congenital
Mixed micro and macronodular cirrhosis	15999000	Congenital
Mucopolysaccharidosis, MPS-II	70737009	Congenital
Groove pancreatitis	722871007	Congenital
Growth retardation, mild developmental delay, chronic hepatitis syndrome	774204006	Congenital
Muscle and heart glycogen synthase deficiency	725027004	Congenital
Muscular hypertrophy, hepatomegaly, polyhydramnios syndrome	773584001	Congenital

Navajo neurohepatopathy	784346006	Congenital
Neonatal hepatosplenomegaly	80378000	Congenital
Neonatal sclerosing cholangitis, ichthyosis, hypotrichosis syndrome	724278007	Congenital
Steroid dehydrogenase deficiency and dental anomaly syndrome	723583009	Congenital
Hepatic encephalopathy in fulminant hepatic failure	449901005	Metabolic
Joubert syndrome with congenital hepatic fibrosis	721847002	Fibrosis
Joubert syndrome with congenital hepatic fibrosis	721847002	Fibrosis
Joubert syndrome with congenital hepatic fibrosis	721847002	Congenital
Neonatal diabetes, congenital hypothyroidism, congenital glaucoma, hepatic fibrosis, polycystic kidney syndrome	724094005	Congenital
Deficiency of alpha-dextrin endo-1,6-alpha-glucosidase	367406009	Metabolic
Copper storage associated hepatitis	370492003	Metabolic
End stage liver disease	708248004	Metabolic
Fulminant hepatic failure	235884008	Metabolic
Fulminant hepatitis	427044009	Metabolic
Gallstone chronic pancreatitis	235951009	Metabolic
Generalised glycogenosis	267424007	Metabolic
Glissonian cirrhosis	536002	Metabolic
Glucose transport defect	237966006	Metabolic
Glucose-6-phosphate transport defect	30102006	Metabolic
Glycogen phosphorylase kinase deficiency	235908005	Metabolic
Glycogen phosphorylase kinase deficiency, autosomal recessive	297252005	Metabolic
Glycogen phosphorylase kinase deficiency, X-linked	297251003	Metabolic
Glycogen storage disease	29633007	Metabolic
Glycogen storage disease due to acid maltase deficiency	274864009	Metabolic
Glycogen storage disease due to acid maltase deficiency, infantile onset	722302009	Metabolic
Glycogen storage disease due to acid maltase deficiency, late-onset	722343009	Metabolic
Glycogen storage disease due to muscle phosphorylase kinase deficiency	819953000	Metabolic
Glycogen storage disease due to muscle pyruvate kinase deficiency	860858001	Metabolic
Glycogen storage disease type Ia	444707001	Metabolic
Glycogen storage disease type III	66937008	Metabolic
Glycogen storage disease type IXB	860860004	Metabolic
Glycogen storage disease type VIII	41527003	Metabolic
Glycogen storage disease type X	37666005	Metabolic
Glycogen storage disease with severe cardiomyopathy due to glycogenin deficiency	717821004	Metabolic
Glycogen storage disease, hepatic form	6075009	Metabolic
Glycogen storage disease, muscular form	15978003	Metabolic
Glycogen storage disease, type I	7265005	Metabolic
Glycogen storage disease, type IV	11179002	Metabolic
Glycogen storage disease, type V	55912009	Metabolic
Glycogen storage disease, type VI	29291001	Metabolic
Glycogen storage disease, type VII	89597008	Metabolic
Glycogen synthase deficiency	237964009	Metabolic
Glycogenosis with glucoaminophosphaturia	61598006	Metabolic
Hepatoencephalopathy due to combined oxidative phosphorylation defect type 1	764962002	Metabolic
Hepatogenous chronic copper poisoning	73475009	Metabolic
Hepatorenal syndrome	51292008	Metabolic
Hepatorenal syndrome as a complication of care	213231008	Metabolic
Hepatorenal syndrome due to a procedure	31005002	Metabolic
Hepatorenal syndrome following delivery	22846003	Metabolic
Hepatosplenomegaly	36760000	Metabolic
Metabolic and genetic disorder affecting the liver	235903001	Metabolic
Hepatic and muscle glycogen phosphorylase kinase deficiency	297254006	Metabolic
Hepatic failure	59927004	Metabolic
Hepatic failure as a complication of care	213230009	Metabolic
Hepatic failure due to a procedure	22508003	Metabolic
Ichthyosis congenita with biliary atresia	235916001	Metabolic
Idiopathic chronic pancreatitis	235953007	Metabolic
Obstructive chronic pancreatitis	235954001	Metabolic
Periodontitis co-occurrent with glycogen storage disease	709561006	Metabolic
Portal hypertension due to cystic fibrosis	707420003	Metabolic
Posthepatitic cirrhosis	27156006	Metabolic
Postnecrotic cirrhosis	86454000	Metabolic
Primary biliary cholangitis	31712002	Metabolic
Primary sclerosing cholangitis	197441003	Metabolic
Pulmonary interstitial glycogenosis	707551007	Metabolic
Phosphate transport defect	237965005	Metabolic
Portal hypertension	34742003	Metabolic
Toxic liver disease with cholestasis	197355005	Metabolic
Wilson's disease	88518009	Metabolic
Zieve's syndrome	44047000	Metabolic
Neonatal diabetes, congenital hypothyroidism, congenital glaucoma, hepatic fibrosis, polycystic kidney syndrome	724094005	Congenital
Renal hepatic pancreatic dysplasia	763891005	Congenital
Renal hepatic pancreatic dysplasia	763891005	Congenital
Restrictive cardiomyopathy secondary to haemochromatosis	56941009	Metabolic
Hepatic coma	72836002	Metabolic
Restrictive cardiomyopathy secondary to haemochromatosis	56941009	Metabolic
Viral hepatitis A with hepatic coma	16060001	Infective
Viral hepatitis A with hepatic coma	16060001	Infective
Viral hepatitis A with hepatic coma	16060001	Infective
Hepatic vein thrombosis	38739001	Metabolic

Viral hepatitis B with hepatic coma	26206000	Infective
Viral hepatitis B with hepatic coma	26206000	Infective
Viral hepatitis B with hepatic coma	26206000	Infective
Viral hepatitis with hepatic coma	40946000	Infective
Viral hepatitis with hepatic coma	40946000	Infective
Viral hepatitis with hepatic coma	40946000	Infective
Drug-induced chronic hepatitis	235889003	Drug_induced
Drug-induced chronic pancreatitis	235955000	Drug_induced
Drug-induced cirrhosis of liver	425413006	Drug_induced
Portal hypertension caused by antiretroviral drug	713542007	Drug_induced
Steatosis of liver caused by retroviral protease inhibitor	713529007	Drug_induced
[X]Chronic viral hepatitis, unspecified	187435007	Infective
[X]Chronic viral hepatitis, unspecified	419541000000109	Infective
[X]Chronic viral hepatitis, unspecified	432261000000109	Infective
[X]Other and unspecified cirrhosis of liver	197553002	Cirrhosis
[X]Other and unspecified cirrhosis of liver	470971000000107	Cirrhosis
3-Beta-hydroxy-delta-5-C27-steroid dehydrogenase deficiency	238033007	Metabolic
Accelerated rejection of liver transplant	432777003	Transplant
Acquired portal-systemic shunt due to cirrhosis	128072003	Cirrhosis
Acute on chronic alcoholic liver disease	713370005	Alcoholic
Acute rejection of liver transplant	431222008	Transplant
Adult polyglucosan body disease	721099001	Metabolic
Advanced cirrhosis	123717006	Cirrhosis
Aftercare for liver transplant done	96601000119101	Transplant
Agenesis of bile duct	1003567000	Congenital
Alcoholic cirrhosis	420054005	Alcoholic
Alcoholic fibrosis and sclerosis of liver	235880004	Alcoholic
Alcoholic hepatic failure	235881000	Alcoholic
Alcoholic hepatitis	235875008	Alcoholic
Alcoholic liver damage	41309000	Alcoholic
Alcoholic steatohepatitis	1234778004	Alcoholic
Alcoholic steatosis	50325005	Alcoholic
Alcohol-induced chronic pancreatitis	235952002	Alcoholic
Alpha-1-antitrypsin deficiency	30188007	Metabolic
Alpha-1-antitrypsin hepatitis	190944000	Metabolic
Alpha-methylacyl-CoA racemase deficiency disorder	700463002	Metabolic
Antichymotrypsin deficiency-alpha-1	235909002	Metabolic
Arteriohepatic dysplasia	31742004	Congenital
Ascites due to alcoholic cirrhosis	1082601000119100	Alcoholic
Atresia of hepatic ducts	204782009	Congenital
Autoantibody negative autoimmune hepatitis	1197704005	Autoimmune
Autoimmune hepatitis	408335007	Autoimmune
Autoimmune hepatitis type 1	721711009	Autoimmune
Autoimmune hepatitis type 2	721712002	Autoimmune
Autoimmune hepatitis type 3	721713007	Autoimmune
Autoimmune liver disease	235890007	Autoimmune
Awaiting transplantation of liver	698305006	Transplant
Benign intrahepatic cholestasis type 1	838305005	Autoimmune
Benign intrahepatic cholestasis type 2	1186854003	Autoimmune
Benign recurrent intrahepatic cholestasis	31155007	Autoimmune
Bile acid CoA ligase deficiency and defective amidation	717047007	Metabolic
Biliary atresia with splenic malformation syndrome	717156002	Congenital
Biliary cirrhosis	1761006	Cirrhosis
Biliary cirrhosis NOS	197311004	Cirrhosis
Biliary cirrhosis NOS	589561000000100	Cirrhosis
Biliary cirrhosis of children	197310003	Cirrhosis
Boichis syndrome	717187000	Congenital
Bronze cirrhosis	399126000	Cirrhosis
Calcific chronic pancreatitis	301009006	Alcoholic
Capsular portal cirrhosis	197296006	Cirrhosis
Cardiac cirrhosis	74669004	Cirrhosis
Cardiac glycogen phosphorylase kinase deficiency	297253000	Metabolic
Cardiac glycogenosis	195025008	Metabolic
Cardiac portal cirrhosis	266470007	Cirrhosis
Cardituberculous cirrhosis	197304003	Cirrhosis
Cholangiolitic cirrhosis	123606000	Cirrhosis
Cholestanol storage disease	63246000	Metabolic
Chorea co-occurrent and due to Wilson disease	724766009	Metabolic
Chronic active hepatitis	197284004	ParentCode
Chronic aggressive hepatitis	197285003	ParentCode
Chronic alcoholic hepatitis	307757001	Alcoholic
Chronic alcoholic liver disease	713181003	Alcoholic
Chronic autoimmune hepatitis	16098491000119100	Autoimmune
Chronic autoimmune hepatitis	16098491000119100	Autoimmune
Chronic fibrosing pancreatitis	74973004	Fibrosis
Chronic hepatic failure	235886005	Metabolic
Chronic hepatic failure due to portosystemic shunt	1197736003	ParentCode
Chronic hepatitis	76783007	ParentCode
Chronic hepatitis NOS	197289009	ParentCode
Chronic hepatitis NOS	589531000000105	ParentCode
Chronic hepatitis unspecified	197288001	ParentCode
Chronic hepatitis unspecified	589521000000108	ParentCode

Chronic liver disease NOS	197323005	ParentCode
Chronic liver disease NOS	592431000000100	ParentCode
Chronic non-A non-B hepatitis	235870003	ParentCode
Chronic persistent type B viral hepatitis	38662009	Infective
Chronic persistent viral hepatitis	60037002	Infective
Chronic rejection of liver transplant	432908002	Transplant
Chronic type B viral hepatitis	61977001	Infective
Chronic viral hepatitis	10295004	Infective
Chronic viral hepatitis B	875211000000105	Infective
Chronic viral hepatitis B with hepatitis D	235869004	Infective
Chronic viral hepatitis B without delta-agent	186639003	Infective
Chronic viral hepatitis D	735451005	Infective
Chronic yellow atrophy of liver	81675001	Metabolic
Cirrhosis of liver NOS	235891006	Cirrhosis
Cirrhosis of liver NOS	662341000000105	Cirrhosis
Cirrhosis of liver not due to alcohol	111370006	Cirrhosis
Congenital absence of cystic duct	890201000	Congenital
Congenital bile acid synthesis defect type 3	719454003	Congenital
Cystic fibrosis related cirrhosis	778131000000103	Cirrhosis
Delta-4-3-oxosteroid-5-beta-reductase deficiency	238035000	Metabolic
Fatal congenital hypertrophic cardiomyopathy due to glycogen storage disease	1230303001	Congenital
Fever-associated acute infantile liver failure syndrome	1208726006	Autoimmune
Glycogen storage disease due to aldolase A deficiency	1187461004	Metabolic
Glycogen storage disease due to lactate dehydrogenase deficiency	1186809004	Metabolic
Glycogen storage disease due to muscle beta-enolase deficiency	1162916008	Metabolic
Glycogen storage disease due to phosphoglycerate kinase 1 deficiency	1187462006	Metabolic
Hepatic ascites co-occurrent with chronic active hepatitis due to toxic liver disease	1092801000119100	Metabolic
Hepatic coma due to viral hepatitis D	1137357005	Infective
Hepatic failure following surgical procedure	1148573000	ParentCode
Hepatic fibrosis due to non-alcoholic fatty liver disease	1197739005	Alcoholic
Hepatitis B and hepatitis C	442374005	Infective
Hepatitis B reinfection following liver transplantation	1230342001	Infective
Hepatitis C genotype 1	824841000000105	Infective
Hepatitis C genotype 2	824851000000108	Infective
Hepatitis C genotype 3	824871000000104	Infective
Hepatitis C genotype 4	824881000000102	Infective
Hepatitis C genotype 5	824891000000100	Infective
Hepatitis C genotype 6	824901000000104	Infective
Hepatitis D superinfection of hepatitis B carrier	235865005	Infective
Hepatitis in late syphilis	197347003	Infective
Hepatitis in secondary syphilis	197348008	Infective
Hypermanganesemia with dystonia, polycythaemia, and cirrhosis	702377007	Metabolic
Idiopathic ductopenia	1234822009	ParentCode
Infectious cirrhosis NOS	197307005	Infective
Infectious cirrhosis NOS	589541000000101	Infective
Isolated neonatal sclerosing cholangitis	1220580006	Congenital
Liver abscess and chronic liver disease causing sequelae NOS	197336005	Infective
Liver abscess and chronic liver disease causing sequelae NOS	622551000000105	Infective
Liver cirrhosis due to classical cystic fibrosis	1010616001	Cirrhosis
Non-alcoholic cirrhosis NOS	266472004	Cirrhosis
Non-alcoholic cirrhosis NOS	671731000000104	Cirrhosis
Oesophageal varices due to cirrhosis of liver	897004000	Cirrhosis
Oesophageal varices due to cirrhosis of liver caused by alcohol	897005004	Alcoholic
Oesophageal varices with bleeding due to primary biliary cholangitis	1197697000	Autoimmune
Other non-alcoholic chronic liver disease	197314007	ParentCode
Other non-alcoholic chronic liver disease	592411000000108	ParentCode
Other non-alcoholic chronic liver disease NOS	197322000	ParentCode
Other non-alcoholic chronic liver disease NOS	592421000000102	ParentCode
Polyglucosan body myopathy type 2	1228849007	Metabolic
Portal cirrhosis	235894003	Cirrhosis
Portal cirrhosis unspecified	197308000	Cirrhosis
Portal cirrhosis unspecified	589551000000103	Cirrhosis
Primary biliary cholangitis and/or primary sclerosing cholangitis and autoimmune hepatitis overlap syndrome	1230291009	Autoimmune
Progressive familial intrahepatic cholestasis type 1	1155913007	Congenital
Progressive familial intrahepatic cholestasis type 2	1155841005	Congenital
Progressive familial intrahepatic cholestasis type 3	1186865008	Congenital
Progressive intrahepatic cholestasis	74162007	Autoimmune
Recurrent hepatitis C virus induced liver disease following liver transplant	1197150002	Infective
Relapsing type A viral hepatitis	43634002	Infective
Secondary portal hypertension	1197664001	ParentCode
Synthetic defect of bile acids	235915002	Metabolic
Westphal-Strumpell syndrome	190823004	Metabolic
